# Screening of attention deficit hyperactivity disorder among preschool children Gharbia Governorate, Egypt

**DOI:** 10.1186/s12888-023-04785-x

**Published:** 2023-04-25

**Authors:** Eman Ali Younis, Safynaz El Saied Shalaby, Sanaa Abd El‐fatah Abdo

**Affiliations:** Department of Public Health & Community Medicine, Faculty of Medicine, Tanta University, Tanta, 31257 Egypt

**Keywords:** ADHD, Screening, Preschool, Egypt

## Abstract

**Background:**

Children who experience mental health issues, such as attention deficit hyperactivity disorder (ADHD), experience significant distress and impairment at home, at school, and in the community. Without adequate care or prevention, this frequently results in adulthood-long distress and impairment at large societal costs. Determining the prevalence of ADHD cases among preschoolers as well as some associated maternal and child risk factors was the aim of this study.

**Methods:**

An analytical cross-sectional study including 1048 preschool children aged 3–6 years was carried out in Tanta City, Gharbia Governorate. From March to April 2022, a proportionate stratified cluster random sample of them was picked. Data were gathered using a predesigned instrument that included sociodemographic information, family history, maternal and child risk factors, and the Arabic version of the ADHD Rating Scale IV questionnaire.

**Results:**

The prevalence of ADHD among preschoolers was 10.5%. The inattention type was the most common (5.3%), followed by the hyperactivity type (3.4%). There were statistically significant associations regarding positive family history of psychological and neurological symptoms (17.9% positive vs. 9.7% negative), family history of ADHD symptoms (24.5% positive vs. 9.4% negative), active smoking by the mother (21.1% positive vs. 5.3% negative), cesarean section delivery (66.4% positive vs. 53.9% negative), elevated blood pressure during pregnancy (19.1% positive vs. 12.4% negative), and history of taking drugs during pregnancy (43.6% positive vs. 31.7% negative). Significant child risk factors were: exposure to any source containing lead that causes slow poisoning (25.5% positive vs. 12.3% negative), children with cardiac health problems (38.2% positive vs. 16.6% negative), and hours spent by a child in front of the TV or mobile phone (any screens) per day (60.0% of those with positive screening spent more than 2 h/day vs. 45.7% negative).

**Conclusion:**

In the Gharbia governorate, 10.5% of preschoolers suffer from ADHD. Significant maternal risk factors for ADHD included a positive family history of psychiatric and neurological symptoms, a family history of ADHD symptoms, active maternal smoking, caesarean section delivery, increased blood pressure during pregnancy, and a history of drug use during pregnancy. Youngsters who had cardiac health issues and who spent more time each day watching TV or using a mobile device (screen use) were at substantial risk.

## Introduction

In children worldwide, attention deficit hyperactivity disorder (ADHD) is one of the most prevalent psychiatric diseases. There are three different varieties of it: largely inattention, hyperactive, and composite type. It is characterized by a concomitant inattention and impulsivity or hyperactivity [1]. Many, interrelated elements, including environmental, developmental, and genetic factors, can contribute to ADHD. In 80% of cases that have been diagnosed, the latter is thought to be the root cause [2].

Also, it is linked to risky prenatal maternal factors that negatively impact the fetus, such as alcohol consumption, stress on the mother, bleeding during pregnancy, corticosteroids, use of illegal drugs, smoking, and exposure to toxins [3]. Research confirmed the link between ADHD and poor intrauterine conditions and came to the conclusion that preterm birth and a small gestational age are related to a higher risk of ADHD [4–6].

In a meta-analysis research published in 2015, the prevalence among people under the age of 18 was estimated to be 7.2% globally, which, according to the 2013 Census Bureau, equates to 129 million youngsters [7, 8].

In the United States National Survey of Children's Health (2016), parents stated that 9.4% of children aged 2 to 17 had been diagnosed with ADHD. More precisely, 2.4% of children aged 2 to 5 had the disorder, 9.6% of children aged 6 to 11 had it, and 13.6% of teenagers aged 12 to 17 had it. Also, they stated that a total of 8.4% of kids between the ages of 2 and 5 years, 8.9% of kids between the ages of 6 and 11 years, and 11.9% of teenagers between the ages of 12 and 17 reported having ADHD now [9].

Prevalence among preschoolers was found to be 4.6% in a 2018 study conducted in Germany [1]. The same year, a research conducted in Egypt revealed a higher prevalence rate of 9.30% among preschoolers [10]. Another study in Egypt from 2014 found that Menoufia schoolchildren had a lower prevalence of ADHD (6.9%) [11].

ADHD often persists into adulthood if not managed and is considered a risk factor for other mental disorders and undesirable outcomes, including educational underachievement, difficulties with employment and interpersonal relations, and criminality [12].

The estimated costs pended on individual adjustment, the reforming of family and school life, health care services, and social services underscore the importance of earlier identification and treatment. To our knowledge, a few studies have investigated the prevalence of ADHD in the Gharbia governorate and its associated risk factors. So, the aim of this study was to determine the prevalence of ADHD among preschoolers and identify some associated maternal and child risk factors in Gharbia Governorate, Egypt.

## Patient and methods

### Study design and settings

This is an analytic cross-sectional study that was conducted in Tanta city, Gharbia governorate, Egypt. Gharbia governorate is in the northern part of Egypt. It consists of 8 cities and covers more than 25 thousand square kilometers, making it the tenth largest governorate in Egypt. Gharbia is also quite densely populated, with more than five million people according to the Central Agency for Public Mobilization and Statistics (CAPMAS). Tanta is Gharbia's capital city and is the fifth most populated city in Egypt, with more than 400 thousand residents.

### Sample size calculation

The sample size was calculated using the Epi Info (2000) program, depending on a past review of the literature. Elsaid N et al. [10] estimated the prevalence of attention deficit hyperactivity disorder among preschool children to be 9.30%; the sample size has been calculated at power 80%, CI 95%, and level of precision of the estimated outcome of 2%. So the sample size calculated was 746 preschool children. We added 20% to compensate for incomplete questionnaires, bringing the total sample size to 870; however, we collected 1048 sheets.

### Sampling technique

A proportionate stratified cluster random sampling technique was used as follows: first stage: simple random by choosing one district (Tanta) out of eight in Gharbia governorate, Egypt. Second stage: stratification of Tanta city into two geographical districts (East and West Tanta Educational Administration) Third stage: stratification of each educational administration into government and private schools The East Administration involved 16 governmental and 9 private schools. The West Administration involved 15 government and 10 private schools. Fourth stage: systemic random selection of schools by list for both governmental and private schools 15 governmental (8 east and 7 west) and 11 private schools (8 east and 3 west) were chosen. Fifth stage: cluster random selections of classes; two classes from each school were chosen randomly (governmental and private classes involved 20/25 and 15/20 preschool students, respectively).

The number of children selected from governmental schools was 563 (298 from the east and 265 from the west administration), representing 64.7% of the total sample size, while the number of children selected from private schools was 307 (205 from the east and 102 from the west administration), representing 35.3% of the total sample size.

**Table:** 

	Total number of schools (50)		Total number of registered children(9372)		Sample size		No schools chosen	
	G	P	G	P	G (563)64.7%	P(307)35.3%	G(15)	P(11)
East Tanta	16	9	3225	2215	298(53%)	205(66.9%)	8	8
West Tanta	15	10	2841	1091	265(47%)	102(33.1%)	7	3

*G* governmental, *P *private

Inclusion criteria: Children ages 3–6 years, both males and females.

Exclusion criteria: children with a neurological or chronic illness, e.g., epilepsy, cerebral palsy, autism, and movement disorders.

Tools of the study: We used two questionnaires.

#### The first questionnaire included the following items

Socio-demographic data include age, sex, residence, birth order, father and mother's education, and family income.

Family history: psychological and neurological illness and history of ADHD among relatives.

Maternal risk factors include neurological and psychological symptoms (convulsions, depression, and stress), active and passive smoking by the mother during pregnancy, timing and type of delivery, blood pressure during child pregnancy, and a history of taking drugs during child pregnancy.

Child risk factors include: child exposure to any source containing lead that causes slow poisoning; significant head injury since birth; cardiac health problems; and the number of hours that the child spends in front of the TV or mobile phone (or any screens) per day.

Healthcare professionals utilize the criteria in the Diagnostic and Statistical Manual, Fifth Edition (DSM-5) of the American Psychiatric Association to diagnose ADHD [13]. With a diligent and comprehensive evaluation, ADHD in preschoolers can be identified with accuracy. These characteristics lead to the following behavioral risk indicators for ADHD in children aged 3 to 4 years, which may aid medical professionals and parents in distinguishing early signs of ADHD from "normal" conduct given that their actions are met with open and unreserved praise [14].

If two or more of the following symptoms are noticed in young children, a referral should be made to a specialist with experience with ADHD in the preschool years [14]. A) Trouble maintaining focus while executing duties B) Quickly loses interest and begins working on another task before completing the previous one. C) Talkative and significantly louder than his fellow college students. D) Often climbs in unsuitable circumstances E) By the age of four, unable to balance on one foot. F) Often wobbles in the chair. G) Go into perilous circumstances out of fearlessness. H) Adjusts to new people too soon. I) exhibits persistent aggression towards peers. J) Usually patched or injured.

#### The second questionnaire

ADHD Screening Questionnaire: ADHD Rating Scale IV (ADHD-RSIV) Arabic Version is originally based on Diagnostic and Statistical Manual of Mental Disorders, Fourth Edition (DSM-IV) criteria and frequently used in epidemiological studies [13]. It contains questions that correspond to the nine symptoms of inattention and the nine symptoms of hyperactivity and impulsivity in the DSM-IV. The ADHD-RSIV was designed for parents to rate the frequency of a child’s symptoms on a scale of 1 to 4, with 1 = never or rarely, 2 = quite often, 3 = very often, and 4 = clearly more than other young people at this age. Children who scored six and above in both (very often and clearly more than other young people at this age) are considered inattentive; children who reported six and more in both (very often and clearly more than other young people at this age) are considered hyperactive-impulsive. Children were classified as having the combined subtype of ADHD if they met the criteria, i.e., six or more in both the inattention and hyperactivity versions.

### Validity and reliability of the study tools

We used a validated and reliable ADHD screening questionnaire that had been used in previous studies [3]. We translated it into Arabic and again into English according to WHO double-translation recommendations by English language experts. The validity of the questionnaire that assesses risk factors was conducted by three Egyptian professors from the public health department at Tanta University faculty of medicine.

Authors tested the risk factors questionnaire reliability in a pilot study by recruiting 20 parents of preschool child not included in the present study. We used data to assess internal consistency using Cronbach’s alpha, which was 0.793 and represented adequate internal consistency.

The authors calculated the content validity index (CVI) and content validity ratio (CVR) as measurements of the content validity of the first questionnaire. The individual-CVI ranged from 0.82 to 1.00, with twenty items having an I-CVI of 1.00 and two items having an I-CVI of 0.82. All items were considered relevant. The CVR was generated for each item. Twenty items had a CVR of 1.00, and two had a score of 0.99.

### Data collection

The study was conducted on 1048 preschool children aged 3–6 years old. They were chosen randomly from selected schools during the period from March 2022 to April 2022. This study was conducted after its approval by the ethical committee of the faculty of medicine at Tanta University. All parents or legal guardians of children gave valid written informed consent after a clear explanation of the study's aims and techniques.

The questionnaires were filled out by parents only or legal guardians in the absence of parents. Parents were asked to fill out a predesigned, self-administered questionnaire and the Arabic form of the ADHD screening sheet. Those who were illiterate were interviewed by the researchers and well-trained third-year medical students during the arrival or departure of their children from school. Children with positive screening results were referred to a psychiatrist specializing in ADHD diagnosis for further assessment and treatment.

### Statistical analysis

Data was collected and statistically analyzed using an IBM personal computer with Statistical Package for Social Science (SPSS) version 21. Qualitative data was expressed as a number and percent and tested by the chi-squared test. Quantitative data was expressed as mean and standard deviation and tested by the t-test. The crude and adjusted odds ratios using logistic regression analysis were calculated at 95% confidence intervals. Logistic regression was used to calculate the effects of risk factors as independent variables. The *P* value was set to be significant at < 0.05.

## Results

**Fig. 1:**
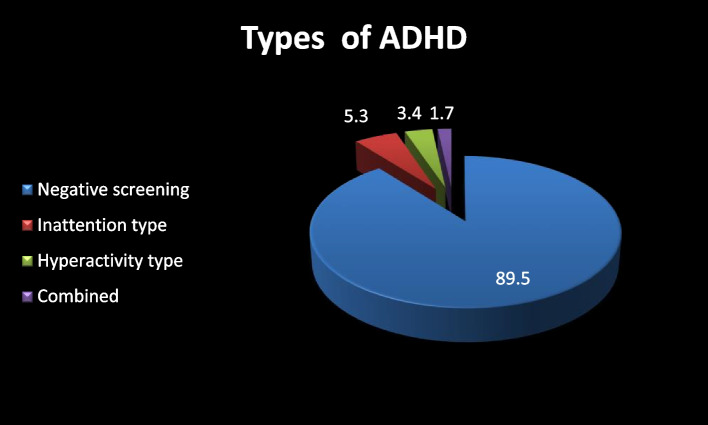
Types of ADHD

The prevalence of attention deficit hyperactivity disorder among preschool children was 10.5%. The inattention type was the most common, present in 5.3% of cases, followed by the hyperactivity type (3.4%) (Fig. 1).

A total of 1048 parents completed the questionnaire. The mean age was nearly the same among both negative and positive screening groups (4.693 + 0.9641 and 4.528 + 0.9730 respectively). More than half of males screened positive (57.3%), compared to 42.7% of females. Also, most rural preschoolers screened positive (68.2%) compared to 31.8% of urban preschoolers. There were statistically significant associations regarding father, mother education (a higher percentage of positive screening was reported in university-educated parents (55.5%) vs. 4.5% for illiterate fathers and 6.4% for illiterate mothers), and also family income (66.4% of those who had enough income were positive screeners compared to 13.6% who had not enough) (Table 1).

**Table 1:** Socio-demographic characteristics among studied groups

Variables	Studied children (*n* = 1048)				Test of significance	*P* value	OD 95% CI
	**Negative screening (*n* = 938)**		**Positive screening (*n* = 110)**				
	**n**	**%**	**n**	**%**			
**Age**							
Mean ± SD	4.693 ± .9641		4.528 ± .9730		t = 1.660	.097	…………
**Gender**							
Male	523	55.8	63	57.3	χ^2^ = .092	.762	1.064
Female	415	44.2	47	42.7			(.714–1.585)
**Residence**							
Rural	619	66.0	75	68.2	χ^2^ = .211	.646	.906
Urban	319	34.0	35	31.8			(.593–1.383)
**Class**							
kg1(3- < 4 years)	337	36.0	49	44.5	χ^2^ = 3.46	.177	…………
Kg2(4- < 5 years)	359	38.3	39	35.5			
Kg3 (5–6 years)	241	25.7	22	20.0			
**Father education**							
Illiterate	13	1.4	5	4.5	χ^2^ = 16.23	**.003**	…………
Primary	33	3.5	3	2.7			
Preparatory	46	4.9	13	11.8			
Secondary	222	23.7	28	25.5			
University	624	66.5	61	55.5			
**Mother education**							
Illiterate	17	1.8	7	6.4	χ^2^ = 19.5	**.001**	…………
Primary	24	2.6	8	7.3			
Preparatory	70	7.5	12	10.9			
Secondary	236	25.2	22	20.0			
University	591	63.0	61	55.5			
**Family income**							
Enough	623	66.4	73	66.4	χ^2^ = 8.11	**.017**	…………
Not enough	63	6.7	15	13.6			
Enough and save	252	26.9	22	20.0			

t = student t test χ^2^ = chi square test OD = odds ratio CI = confidence interval

There were statistically significant associations regarding positive family history of psychological and neurological symptoms (17.9% positive compared to 9.7% negative), family history of ADHD symptoms (24.5% positive compared to 9.4% negative), all degrees of kinship (7.3%, 10.9%, and 7.3% positive compared to 3.1%, 1.8%, and 2.6% negative in the first, second, and third degree, respectively), active smoking of the mother (21.1% positive compared to 5.3% negative), cesarean section delivery (66.4% positive compared to 53.9% negative), elevated blood pressure during pregnancy (19.1% positive compared to12.4% negative), and history of taking drugs during pregnancy (43.6% positive compared to 31.7% negative). Higher frequency of positive screening was detected with antibiotics, hormonal therapy, and anticoagulants taken during pregnancy (15.8%, 13.2%, and 10.5%, respectively), while higher frequency of negative screening was detected with calcium and multivitamins 39.4% (Table 2).

**Table 2:** Family history and maternal risk factors among studied groups

Variables	Studied children (*n* = 1048)				χ^2^	*P* value	OD 95% CI
	**Negative screening (*n* = 938)**		**Positive screening (*n* = 110)**				
	**n**	**%**	**n**	**%**			
**Family history of psychological and neurological symptoms**							
Yes	91	9.7	19	17.3	6.01	**.014**	1.943
No	847	90.3	91	82.7			(1.133–3.333)
**Family history of ADHD symptoms**							
Yes	88	9.4	27	24.5	23.1	**.000**	3.142
No	850	90.6	83	75.5			(1.932–5.111)
**If yes: Degree of kinship (*n* = 115)**							
1^st^	29	3.1	8	7.3	44.9	**.000**	…………
2^nd^	17	1.8	12	10.9			
3^rd^	24	2.6	8	7.3			
**Mother neurological& psychological symptoms**							
Yes	72	7.7	14	13.0	3.5	.058	1.791
No	866	92.3	94	87.0			(.973–3.299)
**Active smoking of mother**							
Yes	49	5.3	23	21.1	37.9	.**000**	4.814
No	882	94.7	86	78.9			(2.798–8.282)
**Passive smoking of mother**							
Yes	330	35.2	37	33.6	.103	.748	.934
No	608	64.8	73	66.4			(.615–1.418)
**Birth order of child**							
1^st^	324	34.5	47	42.7	3.6	.460	…………
2^nd^	312	33.3	35	31.8			
3^rd^	199	21.2	19	17.3			
4^th^	84	9.0	8	7.3			
5^th^ and above	19	2.0	1	0.9			
**Timing of delivery**							
Full term	721	76.9	78	70.9	2.04	.359	…………
Preterm	115	12.3	16	14.5			
Post term	102	10.9	16	14.5			
**Type of delivery**							
Normal vaginal	371	39.6	22	20.0	19.6	**.000**	…………
Cesarean section	506	53.9	73	66.4			
Complicated with forceps, vaccum	61	6.5	15	13.6			
**Blood pressure during child pregnancy**							
Normal	683	72.8	65	59.1	9.1	**.011**	…………
Low	139	14.8	24	21.8			
High	116	12.4	21	19.1			
**History of taking drugs during child pregnancy**							
Yes	297	31.7	48	43.6	6.3	**.011**	1.671
No	641	68.3	62	56.4			(1.119–2.496)
**If yes: mention (*n* = 345)**							
Antibiotics	27	9.6	6	15.8	17.07	**.048**	…………
Anticoagulants	9	3.2	4	10.5			
Hormonal therapy	19	6.7	5	13.2			
Calcium &Multi vitamins	111	39.4	6	15.8			
Hypoglycemic drugs	16	5.7	0	0.0			
Antihypertensive drugs	17	6.0	3	7.9			
Cortisone& anti-inflammatory	4	1.4	0	0.0			
Sedatives	2	0.7	0	0.0			
Anti-thyroid drugs	1	0.4	0	0.0			
More than one	76	27.0	14	36.8			

χ^2^ = chi square test OD = odds ratio CI = confidence interval

Exposure to any source containing lead that causes slow poisoning (25.5% positive compared to 12.3% negative), children having cardiac health problems (38.2% positive compared to 16.6% negative), and hours spent by a child in front of the TV or mobile phone (any screens) per day were significant risk factors for ADHD (60.0% of those with positive screening spent more than 2 h/day compared to 45.7% negative) (Table 3).

**Table 3:** Child risk factors among studied groups

Variables	Studied children (*n* = 1048)				χ^2^	*P* value	OD 95% CI
	**Negative screening (*n* = 938)**		**Positive screening (*n* = 110)**				
	**n**	**%**	**n**	**%**			
**Is the child exposed to any source containing lead that causes slow poisoning?**							
Yes	115	12.3	28	25.5	14.5	**.000**	2.444
No	823	87.7	82	74.5			(1.525–3.915)
**Has the child been exposed to any significant head injury since birth?**							
Yes	149	15.9	23	20.9	1.8	.178	1.400
No	789	84.1	87	79.1			(.856–2.289)
**Does the child have cardiac health problems?**							
Yes	156	16.6	42	38.2	29.8	**.000**	3.096
No	782	83.4	68	61.8			(2.032–4.718)
**How many hours does the child spend in front of the TV or mobile phone (any screens) per day?**							
Less than 2 h	245	26.1	21	19.1	8.04	**.018**	…………
2 h	264	28.1	23	20.9			
More than 2 h	429	45.7	66	60.0			

χ^2^ = chi square test OD = odds ratio CI = confidence interval

The main predictors for ADHD using logistic regression analysis were family history of psychological and neurological symptoms, family history of ADHD symptoms, active smoking of mother, mother neurological& psychological symptoms, complicated delivery, history of taking drugs during child pregnancy, child with cardiac health problems, hours spent by the child in front of the TV or mobile phone (any screens) per day (Table 4).

**Table 4:** Logistic regression of the most relevant risk factors of attention deficit hyperactivity disorder among studied group

Variables	Reference category	Wald	*P*	Exp(B)	95%CI for EXP(B)	
					**Lower**	**Upper**
**Age**		.574	.449	.801	.452	1.422
**Gender**	Male	.252	.615	.785	.305	2.020
**Residence**	Urban	2.086	.149	.460	.160	1.319
**Class**	KG3	.106	.745	.878	.400	1.927
**Father education**	University	.020	.888	.961	.551	1.675
**Mother education**	University	.084	.772	.921	.526	1.611
**Family income**	Enough& save	2.551	.110	.478	.193	1.182
**Birth order of child**	5^th^ and above	.001	.978	.995	.678	1.460
**Family history of psychological and neurological symptoms**	Yes	4.919	**.027**	3.115	1.141	8.500
**Family history of ADHD symptoms**	Yes	15.039	**.000**	.227	.108	.481
**Active smoking of mother**	Yes	13.654	**.000**	.106	.032	.349
**Mother neurological& psychological symptoms**	Yes	6.478	**.011**	6.668	1.547	28.743
**Type of delivery**	Complicated with forceps, vaccum	4.214	**.040**	.375	.147	.957
**History of taking drugs during child pregnancy**	Yes	3.844	**.050**	15.483	1.001	239.507
**Does the child have cardiac health problems?**	Yes	10.663	**.001**	.419	.248	.706
**How many hours does the child spend in front of the TV or mobile phone (any screens) per day?**	More than 2 h	5.586	**.018**	.497	.279	.888

## Discussion

ADHD is a neurodevelopmental disorder with a common onset in the preschool years of childhood. It is characterized by a persistent pattern of inattention and/or hyperactivity. The present study showed a pooled prevalence of ADHD among preschool children of 10.5%.

This is notably larger than other international studies, which showed a lower prevalence of 2 to 7.8% [15, 16]. It is also higher than the prevalence reported from other regional countries [17, 18]. A systematic review in Arab countries reported that the pooled prevalence of ADHD ranged from 1.3 to 16%, the hyperactive type from 1.4 to 7.8%, and the inattention type between 2.1–2.7% [19]. Egyptian studies as well showed variable prevalence ranging from 3.4% to 21.8% [10, 20–22].

The inattention type was the most common type present in our study, affecting 5.3% of cases, followed by the hyperactivity type (3.4%). In agreement with our results, the systematic review in Africa concluded that the inattentive type was the most predominant, followed by hyperactive-impulsive and the combined type, with a prevalence of 2.95%, 2.77%, and 2.44%, respectively [23]. This was surprisingly different from the results of Hassaan FM 2020, who reported the ADHD combined type as the most predominant type (38%), followed by the ADHD inattention type (34%), and the ADHD hyperactive type (28%) [22].

Variability in ADHD prevalence may be attributed to different research methodologies, a lack of parents' knowledge about ADHD, and it being a multifactorial disorder [24]. Also, COVID-19 may be one of the factors responsible for variability in the prevalence as the problem was intensified by the effect of COVID-19 [25, 26].

The current study revealed a higher prevalence of ADHD in males (57.3%) than females (42.7%). However, the difference was not statistically significant. This finding disagrees with other studies such as Albatti (2017), Hamidzadeh et al. 2020, and Hassaan FM 2020 [17, 18, 22]. Gender differences support the evidence for ADHD having a biological or genetically transmitted etiology [27].

ADHD has also been linked to other socio-demographic factors, like first-born status [28], and this disagrees with our results. Residence is one of these factors, and this study revealed a non-statistically significant higher frequency of ADHD among rural preschoolers (68.2%) than urban preschoolers (31.8%). Unlike Hamidzadeh et al. (2021), who reported a significant high frequency of ADHD among children living in rural areas [17], On the other hand, the prevalence of ADHD in urban areas (52.0%) was higher than in rural areas (48.0%) in another Egyptian study [22]. Cultural divergences between urban and rural communities and the role of the environment stand behind these differences [29].

Statistically significant associations regarding fathers', mothers', and enough family income were detected in this study. Hassaan FM 2020 showed a low frequency of ADHD in children with highly educated parents and a high frequency of ADHD in patients with low socioeconomic status, despite being non-statistically significant [22] which is different than our finding, as we found a high prevalence of ADHD among children with university-educated parents, which can be explained by the fact that mothers with a low educational level have a lower threshold for reporting symptoms less precisely [30].

Lower socioeconomic status with a higher prevalence of ADHD was also reported by Rowland AS, 2018 [31]. However, socioeconomic level was not related to ADHD in other studies [15].

The present study reported that a positive family history of psychological and neurological symptoms, a family history of ADHD symptoms, active smoking by the mother, cesarean section delivery, elevated blood pressure during pregnancy, and a history of taking drugs during pregnancy were significant maternal risk factors for ADHD.

These findings are similar to those reported by Hamidzadeh et al. (2021), who found a significant relationship between a family history of psychological problems and ADHD [17]. Research has also observed a relation between ADHD prevalence and cigarette smoking in the early life of children [32]. A number of studies reported some pregnancy and birth-related risk factors, such as maternal exposure to alcohol, tobacco, and cocaine; viral infections during pregnancy; preeclampsia; maternal anemia; lower serum levels of iron and iodine; and trauma to the abdomen, as being associated with increased risk of ADHD [33]. Also, Robinson LR, 2022, reported a strong association between prenatal maternal neurological symptoms and ADHD [4]. In contrast with Schwenke E. (2018), who didn’t find such an association [5].

This study reported that children with cardiac health problems are a significant risk factor for ADHD. This finding is in line with the results of Hamidzadeh et al. (2021) [17, 34]. Conversely, such a significant relationship was not observed in other studies [35].

Increased screen hours spent in front of a TV or mobile phone per day were significant child risk factors for ADHD. Research has widely discussed the relationship between ADHD and screen time. Children and adolescents diagnosed with ADHD or rated as having attention problems or impulsiveness were found to have a greater rate of screen time [36]. Screen exposure time was above the recommended standard of one hour per day in 80.4% of preschool children with ADHD, and it had a positive correlation with the severity of ADHD and parental stress [37].

This relationship may have become more evident in COVID-19 times, when parents had to be parents and teachers at the same time. It’s expected that they may sometimes allow their children more screen time [38].

Logistic regression in this study showed that active smoking by the mother, history of taking drugs during child pregnancy, and screen time are the most relevant risk factors for ADHD among the studied group.

Pregnant women should be encouraged to quit smoking and avoid exposure to tobacco smoke, as maternal active smoking during pregnancy contributes to the increased risk of ADHD at preschool age [39].

A meta-analysis of 27 articles showed that prenatal exposure to maternal smoking during pregnancy was significantly associated with childhood ADHD after adjusting for parental psychiatric history and socioeconomic status [40].

Drugs during pregnancy can affect the baby's growing brain, leading to intellectual and social problems in childhood. Even mothers who took the pain reliever acetaminophen during pregnancy were at higher risk for ADHD and behavior problems [41]. Another meta-analysis in 2018 on 132.738 pairs of mothers and children revealed a 30% higher risk of ADHD for children with prolonged acetaminophen exposure intrauterine [42].

## Conclusion

In the Gharbia Governorate, attention deficit hyperactivity disorder was relatively common among preschoolers. There were certain maternal risk factors found, such as the mother's active smoking, a caesarean delivery, high blood pressure during pregnancy, and a history of drug use while pregnant. Youngsters who had cardiac health issues and who spent more time each day watching TV or using a mobile device (screen use) were at substantial risk. Also, risk variables included a positive family history of psychiatric and neurological symptoms as well as a family history of ADHD symptoms.

### Recommendations

ADHD screening should be a prerequisite for school entry in all Egyptian schools for the early management of these cases to help them lead a good quality of life.

### Strengths

This study recruited a large sample size of both sexes and residence areas in one of the most densely populated parts of the mid-Delta region of Egypt and targeted young children to allow for early detection and proper management of this important disorder.

### Limitation of the study

1. However, parent reported ADHD diagnosis may include false-positive results, especially for mild, untreated cases. Also, a false diagnosis may be due to a more strict assessment of children’s behavior and a free atmosphere for children at home.
2. The authors could not investigate some other risk factors in this study, such as:
  Alcohol intake is low, as the Egyptian population is mostly Muslim. The Holy Book of the Quran strictly prohibits alcohol use.
  Viral infections, as the Egyptian females usually can't differentiate between bacterial and viral infections, and most of them don't prefer to go to physicians, especially for simple infections.
  Also, breast feeding Egyptian females can't remember the duration of breast feeding, and most of them resort to early weaning practices.

## Data Availability

The datasets generated and analyzed during the current study are not publicly available. However, the datasets are available from the corresponding author on a reasonable request.
